# Community social deprivation and availability of substance use treatment and mutual aid recovery groups

**DOI:** 10.1186/s13011-019-0221-6

**Published:** 2019-08-19

**Authors:** Cory M. Morton

**Affiliations:** Department of Social Work, University of New Hampshire, Pettee Hall 119B, Durham, NH 03824 USA

**Keywords:** Availability, Substance use treatment, Recovery support groups, Social deprivation

## Abstract

**Background:**

The spatial distribution of substance use services impacts their use, with greater access to services associated with more positive outcomes. Findings from availability of primary healthcare indicate service shortages exist in areas characterized by social deprivation. This study investigated whether community social deprivation was associated with a lack of availability of substance use treatment or mutual aid recovery support services.

**Methods:**

This is an ecological analysis investigating the availability of substance use services at a community level in the state of New Hampshire. Several public data sources were combined to represent community social deprivation and availability of substance treatment of mutual aid recovery support groups. Principal components analysis and negative binomial regression were used to test the relationship between community structure and the availability of substance use services.

**Results:**

Community social deprivation was characterized by high rates of poverty, no access to motor vehicles, renter-occupied housing, less than a high school degree, and nonemployment. Communities high in measures of social deprivation were associated with increased availability of both substance use treatment and recovery support services.

**Conclusions:**

Contrary to findings in access to primary healthcare services, social disadvantage was positively related to availability for both types of substance use services. This relationship may reflect the stigma associated with substance use where services associated with stigmatized conditions locate in areas with the least resistance to their presence or be a function of affordability of space. Future research could investigate the relationship between access to services and individual client outcomes.

## Background

The ability to access substance use services has an impact on whether individuals successfully manage a substance use disorder [1, 2]. Treatment for alcohol and substance use disorders is predicated upon keeping individuals engaged and present during an intervention [3, 4]. Positive outcomes such as abstinence from substance use, increased self-efficacy, and decreased criminal justice involvement are related to longer time spent in treatment [5, 6]. Attrition from substance use disorder treatment has been shown to increase as distance increases between the service location and client’s home [7–9]. Having to travel greater than one-mile reduced clients’ chances of completing substance use disorder treatment by 50% in a study completed in Baltimore, MD [1]. While shorter physical distance to services is associated with positive outcomes, availability of some forms of health care resources has been cited as an issue in socially disadvantaged communities [9–11]. However, some research has shown treatment rich communities have higher rates of substance use disorders and drug market activity than areas with little resources for formal substance use treatment [10, 12].

Mutual aid recovery support refers to various self-help groups promoting recovery from substance use disorders, the most well-known being Alcoholics Anonymous (AA) and Narcotics Anonymous (NA). These groups are non-professional, informal, and stress progression through a series of steps to achieve abstinence from substance use [13, 14]. Mutual aid recovery groups are integral parts of the substance use service array in the US, as treatment programs often recommend clients attend mutual aid groups during treatment [8]. While controlled research is difficult because of the anonymous, non-professional nature of this treatment modality, outcomes have generally been seen as positive [5, 15].

There has been little attention paid to the availability of mutual aid groups. While no studies have investigated the socioeconomic correlates of the availability of mutual aid groups, one study investigated the role that the location of mutual aid groups played in formal substance use treatment outcomes. Stahler et al. [16] found being discharged from inpatient substance use treatment to a neighborhood with a high density of mutual aid groups reduced the likelihood of attending outpatient treatment. So, while participation in mutual aid groups has been shown to be positive for substance use outcomes overall, there is evidence that individuals may use mutual aid groups as a substitute for formal outpatient treatment. In terms of the availability of formal types of substance use treatment, greater metropolitan area concentrations of racial and ethnic minority groups was associated with greater availability of methadone treatment but lower availability of comprehensive substance use assessment services and naltrexone treatment in a study using the National Survey of Substance Abuse Treatment Services [17].

This study explores the link between measures of social deprivation and the availability of two forms of substance use services: formal treatment and mutual aid recovery support groups. Prior work in access to health care and substance use treatment have shown poverty and social disadvantage as strong predictors of a lack of available health care resources [9, 11]. This study adds to the literature by testing whether the spatial distribution of substance use treatment mirrors findings in primary care and extending this work to the availability of mutual aid recovery support groups.

## Methods

Data for this study were collected from several sources to represent the substance use treatment and recovery support environment in New Hampshire, a state in the Northeastern United States. According to the National Survey on Drug Use and Health [17], New Hampshire (NH) has some of the highest rates of substance use and substance use disorders in the US. NH ranks in the top 10 nationally for the percentage of residents over 18 engaging in past month binge drinking (30.0), illicit drug use (13.8), and marijuana use (12.4) [18]. Additionally, NH had the highest percentage of adults with past year heroin use (.9) in the US and the fifth highest percentage of adults with an illicit drug use disorder (3.4) [18]. The unit of analysis for this study is the census tract, a measure commonly used to proxy neighborhood boundaries [19, 20].

### Measures

#### Substance use treatment resources

Two data sources were cross-referenced to construct a listing of formal substance use treatment resources. The NH Alcohol and Drug Treatment Locator and the NH Board for Licensing Alcohol and Other Drug Use Professionals provided a list of all inpatient and outpatient substance use treatment locations in the state of New Hampshire. These listings were cross-referenced to remove duplicates. The address of each facility was then geocoded using ArcGIS 10.3.4. with a 98% match rate and joined to the census tract where they were located. Counts for each census tract were used as an indicator of treatment availability.

#### Mutual aid recovery resources

Four data sources were combined to create a measure of mutual aid resources. Statewide listings of meeting locations for Alcoholics Anonymous, Heroin Anonymous, Narcotics Anonymous, and NH Self Management and Recovery Training (SMART) were cross-referenced to compile a listing of mutual aid recovery support locations. The address of each was geocoded (99% match rate) and following the process above, counts in each census tract were calculated as an indicator of mutual aid availability.

#### Social deprivation indictors

The US Census’ 2016 American Community Survey 5-year estimates provided data on the sociodemographic indicators that make up the Social Deprivation Index (SDI) at the census tract level [11]. Indicators included: less than a high school degree (% of residents over the age of 25 with no high school diploma); residential crowding (percentage of housing units with more people than rooms); percentage of residents with no available motor vehicle, nonemployed (percentage of individuals in a census tract that are unemployed and those not participating in the labor force [21]); percentage of residents with income below the federal poverty line; percentage of renter-occupied housing units; and percentage of single headed households.

### Analytic strategy

Following the strategy used by Butler et al. [11], a Social Deprivation Index was created by performing principal components factor analysis (PCA) on the set of social deprivation indicators. Factors with minimum loadings of .6 and maximum cross loadings of .3 were retained. Each of the census indicators was first transformed to its centile ranking before completing the PCA to have the variables represent a common scale [7]. The factor loadings were then used in a negative binomial regression to predict the association of neighborhood level social deprivation and availability of substance use services, either formal substance use treatment or mutual aid recovery support. A negative binomial regression was employed to account for the overdispersion in the dependent variables, where the variance is greater than the mean [22]. This overdispersion occurs here because many census tracts have zero counts for each of the dependent variables. The regression will control for the rurality of a census tract and the percentage of residents that are uninsured. Since the data are spatially ordered, dependence between the units of observation was tested using the Durbin Watson statistic which tests for correlation between the residual errors in a regression, values between 1.8 and 2.0 indicate no autocorrelation [23, 24].

## Results

Descriptive statistics for the variables used in the analysis are presented in Table 1. The principal components factor analysis revealed one component to describe social deprivation which explained 60% of the variance in the set of variables. Single-headed households had high cross-loadings (>.3) and was subsequently removed from the model. Table 2 presents the factor loadings for the initial and reduced set of variables used to create factor loading values. Social disadvantage was characterized by high percentages of poverty, renter occupied housing, having less than a high school degree, nonemployment, and not owning a car.

**Table 1:** Descriptive statistics for social deprivation indicators and substance use service availability

Variable	Mean	Standard Deviation
Social Deprivation Indicators (%)		
Less than high school graduate	7.62	4.98
Residential Crowding	.45	.84
No motor vehicle	2.22	3.33
Renter-occupied housing	28.37	21.32
Nonemployed	22.92	7.65
Single headed households	7.54	3.89
Poverty	9.32	7.49
Substance Use Service Availability	Median	Interquartile Range
Substance use treatment facilities (*n* = 242)	0	0–1
Mutual aid recovery support facilities (*n* = 436)	1	0–2

Note: Unit of measurement is census tracts (*N* = 292)

**Table 2:** Factor loadings of social deprivation indicators

	Factor Loadings	
Variables	Initial	Reduced
Residential Crowding	.224	
Poverty	.900	.903
No motor vehicle	.699	.696
Renter occupied housing	.769	.772
Less than high school degree	.738	.741
Nonemployed	.728	.732

Note: Unit of analysis census tract (*N* = 292)

Table 3 present the findings of the two negative binomial regressions for availability of substance use treatment and mutual aid recovery support. The Durbin Watson statistic was 2.0, indicating spatial autocorrelation was not creating too much noise in the model. In the substance use treatment availability model, social deprivation was positively related to availability while rurality was negatively related to availability. Formal substance use treatment facilities were more likely to be located in census tracts that were less rural. In the mutual aid recovery support model, social deprivation was positively related to availability.

**Table 3:** Negative binomial regression of substance use service availability and social deprivation

	Substance Use Treatment Availability		Mutual Aid Recovery Support Availability	
	β	*SE*	β	*SE*
Social Deprivation	.530***	.132	.441***	.110
Rural	−.011***	.003	.000	.002
Uninsured	−.006	.029	−.010	.024

Note: Treatment Model, Likelihood ratio chi square = 73.44***; Durbin Watson = 2.01 Mutual Aid Model, Likelihood ratio chi square = 30.97***; Durbin Watson = 2.00

***p < .001

## Discussion

The findings from this study indicate both substance use treatment and mutual aid recovery support groups are concentrated in socially disadvantaged neighborhoods. The relationship was stronger for formal substance use treatment services. These findings differ from findings on the availability of physical health care access, where neighborhoods high on social disadvantage had limited access to primary care services [11]. Attention to where substance use treatment facilities are located is important as Archibald [25] found rates of substance use disorder increased in communities with a high density of treatment providers. The need for substance use treatment appears equally distributed along the continuum of high- to low-socially disadvantaged communities, with some differences on substance of choice [26, 27]. While alcohol and marijuana use were more prevalent in high income communities [26], heroin use has seen increases among all income brackets in the US [27]. If the presence of substance use services in a community is not driven by need, what are the main drivers of the decisions to locate in a particular area? The simplest answer is a function of cost, formal substance treatment facilities may locate in areas with lower overhead costs. However, it is possible these findings represent a function of the stigma surrounding substance use where service facilities locate in areas with the least community resistance to their presence. Results here provide a baseline measure of substance use service availability as the state of NH moves to integrate physical and behavioral health services [28]. If substance use treatment migrates to primary care clinics, there could be dramatic shifts in the socioeconomic profile of communities rich in options to manage a substance use disorder.

This study is limited by its cross-sectional nature and focus on availability of services and not how individuals access those services. Results would be strengthened to determine the temporal effect, if any, of how fluctuations in neighborhood level disadvantage impact availability of substance use services and how accessibility functions for individual clients. Stahler et al. [16] found neighborhood density of mutual aid recovery support groups had a negative relationship with outpatient treatment attendance, but it is unknown why this occurs. Did clients find mutual aid to be effective as a treatment modality or was ease of access or affordability more important? The study is also limited by its focus on a single state in the Northeastern US, results here may not translate to states outside of the New England area.

Additional research may consider the relationship between treatment service density and client outcomes. While some studies find reduced distance to treatment predicts more positive treatment outcomes, others have found a more nuanced relationship where treatment facility density can be considered a trigger to substance use or relapse [6, 29]. Where treatment is scarce, clients may see markedly different conditions between their home neighborhoods and where their treatment is located. Jacobson [6] found about one-fifth of clients were exposed to more drug market activity while attending treatment and Kao et al. [29] found clients in treatment dense neighborhoods expressed more anxiety about future heroin use. These relationships may be different for mutual aid density as these groups often operate in a less public fashion (i.e., co-located with a church or community center) than formal treatment centers who have public signage and often advertise their services in a community.

## Conclusions

The availability of substance abuse treatment and mutual aid recovery support services was higher in census tracts characterized by indicators of social deprivation. These findings run counter to investigations of the availability of primary healthcare services [11]. The findings here support more research to fill in the gaps in knowledge around the effect place has on the delivery of substance use services and what drives the locational strategies of treatment and recovery support services.

## Data Availability

The data generated/analyzed during the current study are available from the corresponding author on reasonable request.

## Abbreviations

AA: Alcoholics anonymous
MD: Maryland
NA: Narcotics anonymous
NH: New Hampshire
PCA: Principal components analysis
SDI: Social deprivation index
SE: Standard error
SMART: Self-Management and Recovery Training
US: United States
β: Beta
